# Large acute epidural hematoma from head pin fixation fracture

**DOI:** 10.1002/ccr3.9210

**Published:** 2024-07-19

**Authors:** Vinicius Trindade Gomes da Silva, Louise Makarem, Rhuann Pontes dos Santos Silva, Manoel Jacobsen Teixeira, Wellingson Silva Paiva

**Affiliations:** ^1^ Division of Neurosurgery at University of São Paulo São Paulo Brazil; ^2^ Catholic University of Pernambuco Recife Brazil

**Keywords:** brain tumors, cranial epidural hematoma, fixation fracture, head pin fixation, postoperative complications

## Abstract

Regarding head immobilization practices in neurosurgery, secondary fixation fractures are rare, underscoring the importance of precise pin positioning and an adequate force in the three‐point clamp to achieve adequate fixation. Attention should be given to factors such as changes in bone metabolism.

## 1 INTRODUCTION

Head immobilization, achieved through pin fixation, is a common practice in neurosurgery.^1^ This method ensures stable fixation of the head and neck during intracranial and spinal surgeries, enabling the application of safe and efficient microsurgical techniques. More recently, it has facilitated the use of frameless neuronavigation.^2^ Consequently, intraoperative repositioning of the head becomes feasible, while mitigating the risk of skin damage that may occur when the face rests against a padded head support for extended periods.^3^

Among the commercially available cranial fixation pin systems are the Mayfield Skull Clamp (Integra NeuroSciences, Plainsboro, NJ) and the Sugita Head Holder (Mizuho Ikakogyo Co., Ltd., Tokyo, Japan). The Sugita Head Holder, designed as an innovative microneurosurgical assembly, consists of a head holder with an attached frame to secure flexible self‐retaining retractors (Leyla‐Yasargil), hand rests, microsurgical instrument holders, a plate for cotton patties, and skin‐flap spring retractors.^4^

Despite the widespread use of such devices, associated complications are exceedingly rare and may include head slippage, traumatic aneurysm of the superficial temporal artery, infection at the scalp perforation site, venous air embolism, tension pneumocephalus, penetration into the skull, and epidural hematoma.^5^, ^6^ This discussion focuses on a description of an epidural hematoma as a complication arising from pin fixation.

## 2 CASE HISTORY/EXAMINATION

**FIGURE 1:**
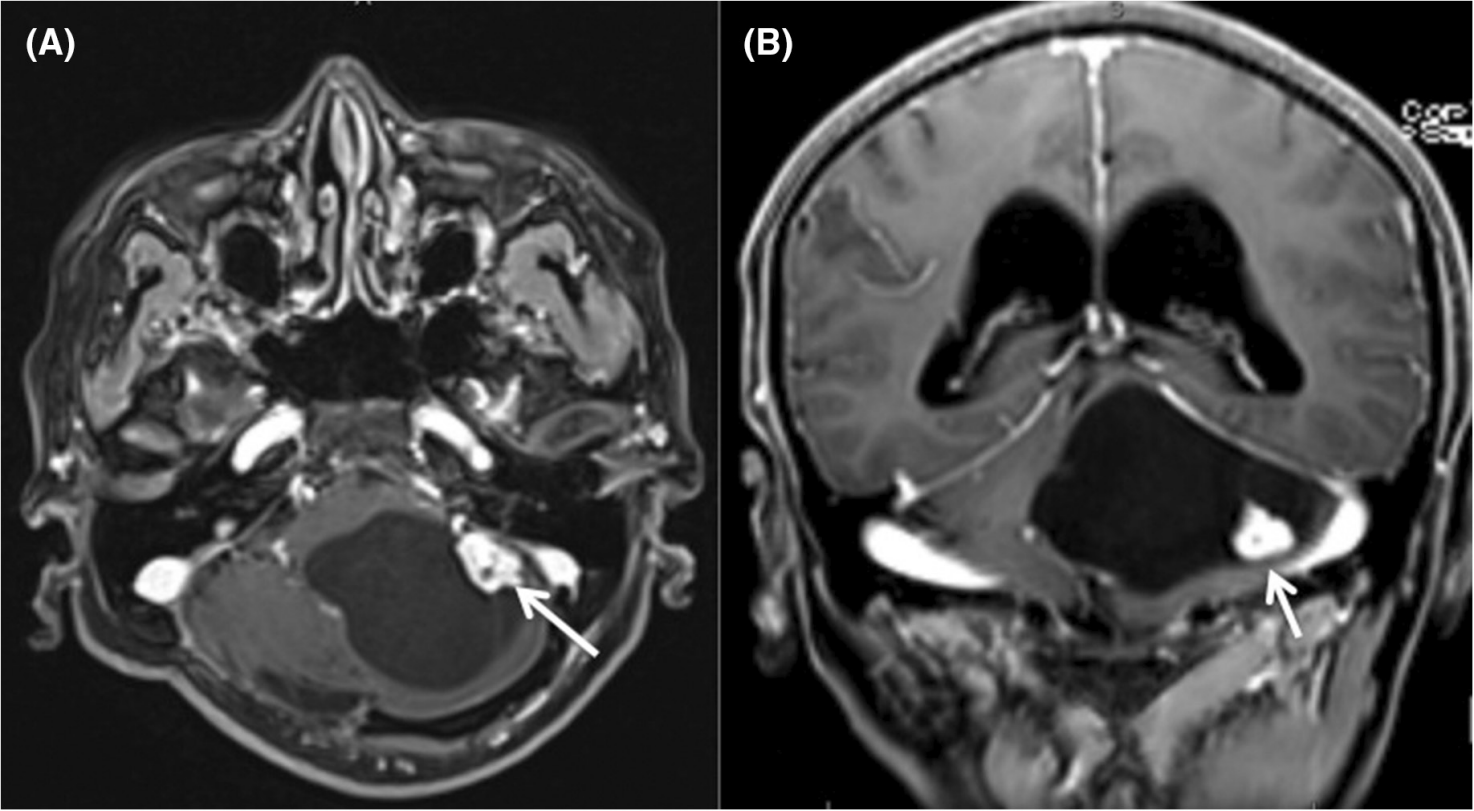
Intense contrast uptake lesion (arrow) and bulky cystic component suggesting hemangioblastoma in a patient with Von Hippel–Lindau syndrome. (A) Magnetic ressonance image in axial plane. (B) Magnetic ressonance image in coronal plane.

A 48‐year‐old female patient was diagnosed with Von Hippel Lindau syndrome. She had a history of bilateral pheochromocytoma resection in 1983, right cerebellar hemangioblastoma surgery in 1992, and resection of a neuroendocrine tumor of the pancreas in 2006. Two months prior to presentation, she developed a global cerebellar syndrome, and neuroimaging revealed a new left cerebellar lesion (Figure 1).

## 3 METHODS

**FIGURE 2:**
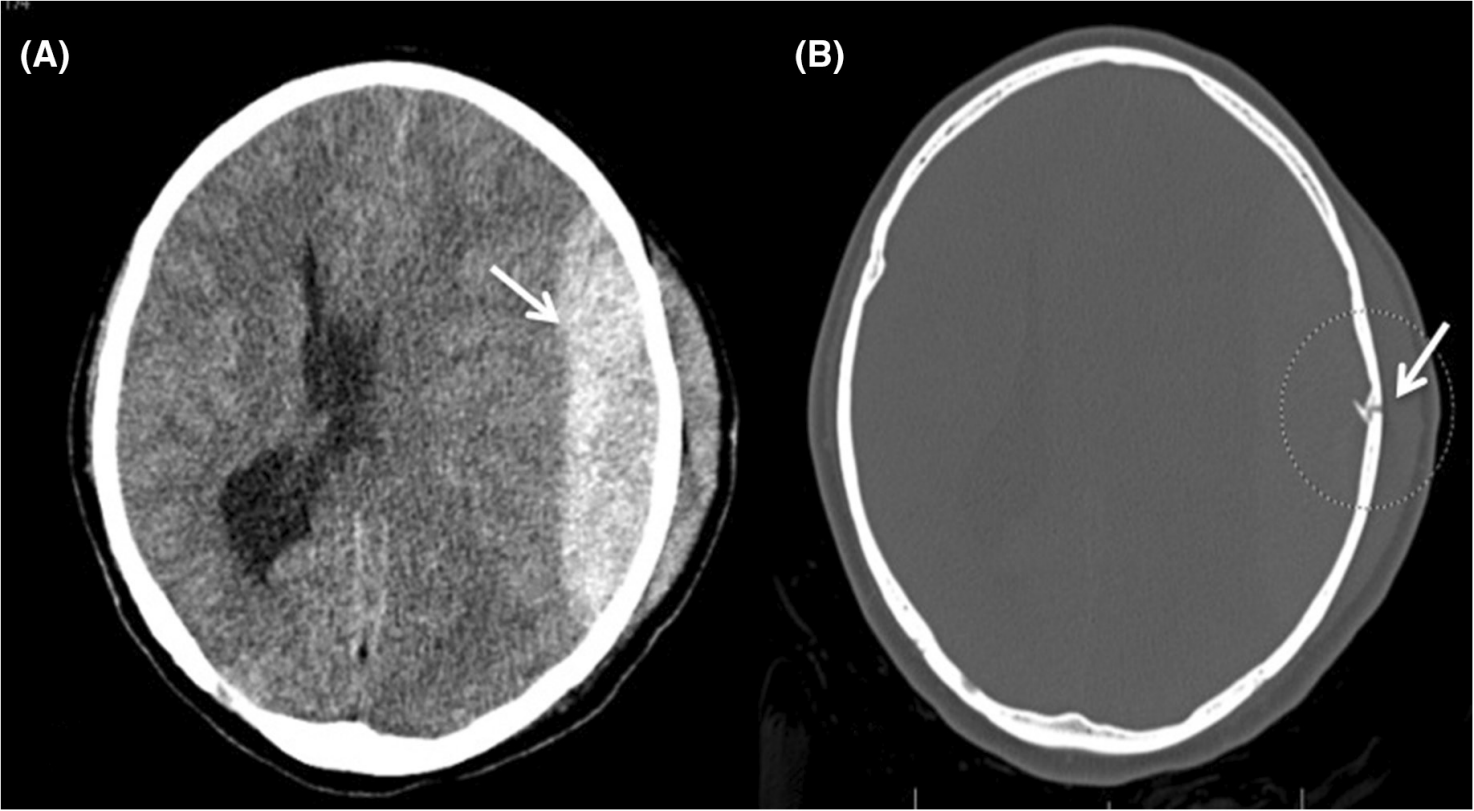
(A) Postoperative skull computed tomography showing large acute epidural hematoma. (B) Computed tomography with bone window showing fracture related to head fixating pin.

We performed a posterior fossa craniotomy for tumor debulking and complete resection using a three‐point head fixation system, the Sugita Head Holder, without any complications. Immediately after the surgery, she developed fixed pupils. A skull computed tomography (CT) scan showed a large fronto‐temporo‐parietal epidural hematoma with midline shift, attributed to a head pin fracture (Figure 2).

## 4 CONCLUSION AND RESULTS

**FIGURE 3:**
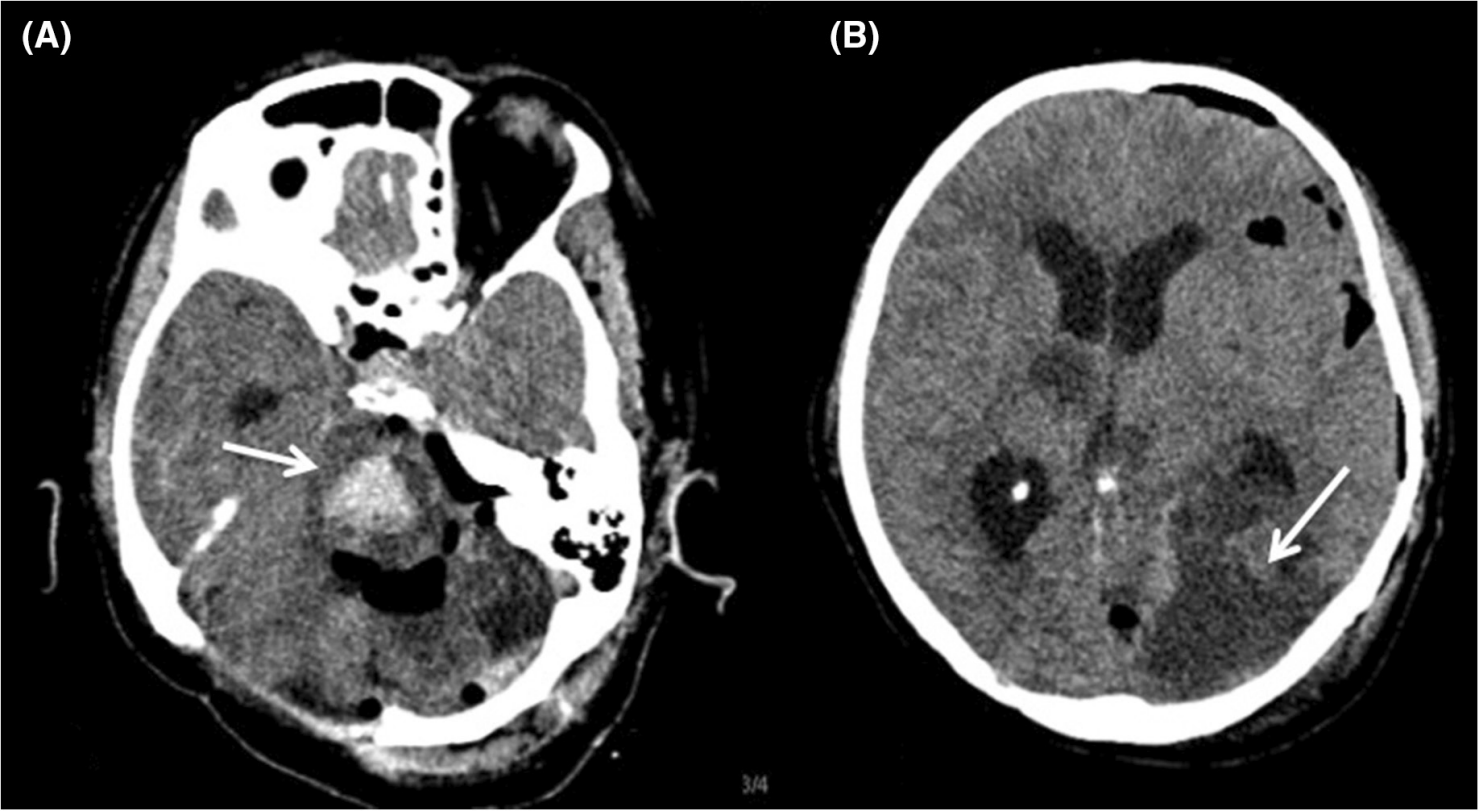
Skull computed tomography performed after epidural hematoma evacuation. (A) Brainstem hematoma. (B) Occipital ischemia suggesting brain herniation.

The patient underwent hematoma evacuation, but subsequent CT scans revealed a brainstem hematoma and ischemic areas (Figure 3). She remained comatose and passed away 2 months later.

Secondary fixation fractures are rare, underscoring the importance of precise pin positioning while avoiding fracture‐prone areas, paranasal sinuses, and venous sinuses. In adult patients, a force of 60–80 lb is applied across the three‐point clamp to achieve adequate fixation. Attention should be given to factors such as changes in bone metabolism, including osteoporosis, chronic kidney disease, and the chronic use of steroids. This raises concerns regarding head immobilization practices in neurosurgery.

## 5 DISCUSSION

For most intracranial procedures, secure cranial fixation is essential. A pin‐type head holder represents the optimal means of achieving the required stability.^3^ It is a frequently employed device in neurosurgery, offering both stability and flexibility in head immobilization.^4^

The Sugita multipurpose head frame ranks among the most commonly utilized head holders in neurosurgery, offering certain advantages over other pin‐based head holders. Its four‐prong pin system reduces the likelihood of slippage; however, the sharp‐pointed pins and a rotational fixation mechanism, as opposed to simple pressure, may potentially elevate the risk of certain complications.^3^

Complications associated with three‐point skull clamps have been reported, including depressed skull fractures, middle meningeal arteriovenous fistulas, venous air embolisms, and epidural hematomas.^7^ Skull fractures and accompanying intracranial hemorrhages are more prevalent among pediatric patients due to the relatively thinner nature of their skulls.^8^ In contrast, in normal adult patients, skull fractures and hematomas resulting from three‐pronged head clamps are exceedingly rare.^9^ In fact, Palmer et al. (1994)^10^ reported a post‐operative epidural hematoma incidence of 0.3% in a cohort of 6668 patients, with none of these cases attributed to the pin headrest. Intracranial pathologies causing sustained increased intracranial pressures and hydrocephalus may lead to skull thinning, heightening the risk of injury associated with pin fixation.^9^

Penetrating skull injuries due to pin headrest devices are primarily observed in children.^11^ In a study conducted by Vitali and Steinbok (2008),^12^ five out of 766 children (0.65%), who underwent craniotomies with pin fixation experienced depressed skull fractures and/or epidural hematomas resulting from the pin fixation. In their case series, the authors correlated these complications with factors such as the presence of a posterior fossa tumor, temporal pin application, extended surgery duration, the presence of hydrocephalus, and an age below 7 years.^12^

Epidural hematomas pose a risk of mortality and acquired neurological impairment.^1^, ^13^, ^14^ Epidural hematomas secondary to pin fractures are infrequent, with a higher prevalence in children. In 2008, Vitali published a series of five cases of fractures in a sample of 766 children, with four of them developing epidural hematomas.^12^ All children who presented with hematomas had undergone posterior fossa tumor surgery. Yan reported a similar case in 2007, involving a substantial epidural hematoma following tumor debulking in the posterior fossa.^7^

Lang (2001)^15^ reported that the average thickness of the skull in the middle of the parietal bone measured 6.32 mm (ranging from 3.5 to 6.8 mm) in adults. Letts et al. conducted a biomechanical study revealing that bones with a 2 mm thickness could support a pressure of 160 lb.^16^

## AUTHOR CONTRIBUTIONS

**Vinicius Trindade Gomes da Silva:** Conceptualization; data curation. **Louise Makarem:** Formal analysis; investigation; visualization; writing – original draft. **Rhuann Pontes dos Santos Silva:** Methodology. **Manoel Jacobsen Teixeira:** Methodology; project administration; visualization; writing – review and editing. **Wellingson Silva Paiva:** Project administration; visualization; writing – original draft; writing – review and editing.

## FUNDING INFORMATION

None.

## CONFLITS OF INTEREST STATEMENT

The authors have no conflict of interest to declare.

## CONSENT

Written informed consent was obtained from the patient to publish this report in accordance with the journal's patient consent policy.

## Data Availability

None.
